# The association between living alone and health care utilisation in older adults: a retrospective cohort study of electronic health records from a London general practice

**DOI:** 10.1186/s12877-018-0939-4

**Published:** 2018-12-05

**Authors:** Kathryn Dreyer, Adam Steventon, Rebecca Fisher, Sarah R. Deeny

**Affiliations:** The Health Foundation, 90 Long Acre, London, WC2E 9RA UK

**Keywords:** Household, Older, Utilisation, General practice, Emergency care, Inpatient care

## Abstract

**Background:**

In 2016, one in three older people in the UK were living alone. These patients often have complex health needs and require additional clinical and non-clinical support. This study aimed to analyse the association between living alone and health care utilisation in older patients.

**Methods:**

We conducted a retrospective cohort study of 1447 patients over the age of 64, living in 1275 households who were registered at a large general practice in South East London. The utilisation of four different types of health care provision were examined in order to explore the impact of older patients living alone on health care utilisation.

**Results:**

After adjusting for patient demographics and clinical characteristics, living alone was significantly associated with a higher probability of utilising emergency department and general practitioner services, with odds ratios of 1.50 (95% confidence interval [CI] 1.16 to 1.93) and 1.40 (95% CI 1.04 to 1.88) respectively.

**Conclusions:**

Living alone has an impact on health care service utilisation for older patients. We show that general practice data can be used to identify older patients who are living alone, and general practitioners are in a unique position to identify those who could benefit from additional clinical and non-clinical support. Further research is needed to understand the mechanism driving higher utilisation for those patients who live alone.

**Electronic supplementary material:**

The online version of this article (10.1186/s12877-018-0939-4) contains supplementary material, which is available to authorized users.

## Background

In 2016 one in three older people in the UK were living alone, an increase of 16% over two decades [1–3]. To address the complex health needs of older people who are often living with multiple-long term conditions [4], commissioners and clinicians often attempt to identify those at highest risk of hospital admission to target with additional clinical community care and enhanced social support [5–7]. However, there has been limited success in the application of such care models, perhaps because the majority of risk stratification tools use only selected clinical data and do not consider patient preferences or the wider social support a patient may have access to in their home, family or the community [7–9].

Although it is widely accepted that a person’s living arrangements are influential on their health [10–12], few studies have examined the association between living arrangements and health care utilisation. While some existing studies have shown an association between living alone, or households with only older people, and increased health care utilisation [13–17], other UK-based studies have not found the same effect [18, 19]. However, many of these studies rely on survey data or self-reported utilisation [13–15, 18, 19]; analyse patterns at a practice rather than at an individual patient level [16]; or focus on only one element of health care services [13, 15–17]. The results of existing studies provide conflicting evidence of the impact of living alone on health care utilisation, this study adds to the literature by examining the relationship at a patient level using electronic health records.

In this study, we assess the relationship between living alone and primary and secondary care utilisation in the English NHS by using electronic health records from a large multi-site general practice, linked at the individual patient address level. Such datasets are increasingly available for use in quality improvement, thus our findings have widespread applicability. We focus our study on older patients as this population is at particular risk of both hospital admission and isolation.

## Methods

### Dataset

This was a retrospective, observational, cohort study using data from the pseudonymised electronic health records of all 25,252 registered patients (as of September 2015) at a multi-site urban general practice, Valentine Health, in South-East London. The population served by Valentine Health is young and deprived, compared to the English average [20]. Address data is routinely recorded on the electronic health record upon registration at a general practice. Patients registered at the same address were automatically grouped into ‘households’ using accessible address matching software and a unique household identifier was generated for each unique address. The electronic health records included patient demographic information, long-term condition diagnoses, prescriptions, general practice appointments, secondary care utilisation data and a lower super output area (a small geographic area used for reporting small area statistics in England). Secondary care utilisation data were reported by local secondary care providers, and were recorded in the electronic patient records by Valentine Health.

### Inclusion and exclusion criteria

We studied patients registered at the general practice as of 22nd September 2015. Patients were grouped into households, based on the unique household identifier. Our cohort of interest were patients aged over 64 as of 22nd September 2015. Prior to pseudonomysation at the general practice, patients without complete data, including address data, were excluded. To allow for full follow up we excluded patients who died before 21st September 2016. Households with over 5 older people were excluded, as, following a discussion with the lead general practitioner, this was likely to be some form of residential social care and outside the scope of our analysis.

### Outcomes

We examined two main outcomes of interest; the probability of at least one emergency department attendance or at least one inpatient admission, between 22nd September 2015 and 21st September 2016, to evaluate the increased risk of acute care utilisation.

Included in the data was the utilisation of general practice and outpatient care between 22nd September 2015 and 21st September 2016. We examined high users of general practice and outpatient services, as defined by the highest quintile of utilisation, as secondary outcomes. General practice appointments that were recorded as incomplete, either because of cancellation or the patient not arriving, were not included in our analysis.

### Covariates

Our observable covariates were age, gender, socio-economic deprivation, number of physical long-term conditions and the presence of a psychological long-term condition. To determine a patient’s local socio-economic deprivation, we used their lower super output area and ranked the 2010 index of multiple deprivation, a measure of relative deprivation for small areas, scores to produce quintiles (with 1 being the most deprived and 5 the least deprived). Long-term conditions were identified using QOF read codes [21] and were based on active diagnoses in the data set.

### Statistical methods

The association between older patient health care utilisation and living alone was examined using multivariable generalised linear models, using a logit link. We modelled the likelihood of an older person accessing a higher level of services than the identified thresholds of utilisation using a binomial distribution. Two models were run to quantify the association between living alone and health care utilisation: an unadjusted model which only controlled for living alone; and an adjusted model which controlled for relevant patient demographic and clinical characteristics, as well as living alone. Odds ratios were produced to interpret the results.

### Subgroup analysis

A subgroup analysis was conducted to investigate whether the association between living alone and health care utilisation changes as patients age. Three subgroup analyses were performed which limited the cohort to older patients over the age of 69, 74 and 79 respectively.

### Sensitivity analysis

To test the robustness of the model, we changed the utilisation thresholds to determine whether our results were dependent upon our choice of threshold. For general practice and outpatient utilisation a higher threshold, the highest decile of utilisation, was tested as we were most interested in older patients who were high users of care. Due to the small number of patients with any emergency department and inpatient utilisation, we were unable to perform any sensitivity analysis for these types of health care services.

## Results

### Patient characteristics

The analysis cohort comprised 1457 patients over the age of 64, which we defined as older patients. One household with more than five older people was excluded from the analysis.

The final analysis cohort included 1447 older patients, living across 1275 households. 6.3% of all households in the complete patient dataset consisted of older patients living alone, in comparison to 9.3% of households in the borough [22]. 51.2% of older patients lived alone and the average size of a household for older people who didn’t live alone was 3.1 (SD 1.70). Patients living alone were on average older; 28.1% of patients living alone were over the age of 80, while only 18.3% of patients living with others were over the age of 80. 55.7% of older patient’s living alone were female; this was not significantly different from the proportion of women living with others. Furthermore, patients living alone had a higher number of chronic conditions; 49.8% of patients living alone had three or more chronic conditions, in comparison to 42.2% of those living with others – see Table 1.

**Table 1:** Utilisation statistics and patient characteristics by household structure, percentages and mean (standard deviation)

	Household Structure		
	Alone (*n* = 741)	Other (*n* = 706)	Total cohort (*n* = 1447)
Utilisation statistics			
At least 12 general practice appointments (%)	21.2	13.9	17.6
At least 1 emergency department attendances (%)	30.6	20.7	11.2
At least 1 inpatient admissions (%)	25.5	18.7	9.7
At least 5 outpatient appointments (%)	17.8	16.7	17.3
Mean general practice appointments (SD)	7.6 (8.6)	6.2 (5.8)	7.0 (7.4)
Mean emergency department attendances (SD)	0.7 (2.7)	0.3 (0.9)	0.5 (2.0)
Mean inpatient admissions (SD)	0.5 (1.2)	0.3 (0.9)	0.4 (1.1)
Mean outpatient appointments (SD)	2.4 (3.2)	2.2 (6.8)	2.3 (3.1)
Patient characteristics			
Mean age (SD)	74.7 (8.1)	73.1 (6.8)	73.9 (7.5)
Female (%)	55.7	50.8	53.4
Age band (%)			
65 to 69	35.6	38.2	36.9
70 to 74	22.5	24.8	23.6
75 to 79	13.6	18.7	16.1
80 plus	28.2	18.3	23.4
Index of Multiple Deprivation quintile (%)			
1 (most deprived)	18.6	21.4	20.0
2	19.7	19.3	19.5
3	22.0	13.6	17.9
4	17.3	20.0	18.6
5 (least deprived)	22.4	25.8	24.0
Long-term condition count (%)			
0	9.6	13.2	11.3
1	17.1	19.3	18.2
2	23.5	25.4	24.4
3 plus	49.8	42.2	46.1
Mean physical long-term condition count (SD)	2.5 (1.7)	2.2 (1.6)	2.3 (1.7)
Psychological long-term condition (%)	26.2	19.0	22.7

Patients living alone had the highest utilisation across all health care services, indicating a potential higher need for this subgroup of patients. Having either an emergency attendance or an inpatient admission increased the likelihood of a patient being a high user of general practice appointments and outpatient appointments – see Additional file 1: Table S1.

### Statistical analysis

In the unadjusted analysis, we found that patients living alone had a significantly higher probability of utilising general practice, emergency department and inpatient care services. Living alone was not statistically significant when analysing outpatient appointments – see Additional file 2: Table S2.

After adjusting for patient demographic and clinical characteristics, we found that living alone was still significantly associated with a higher probability of utilising emergency department and general practitioner services, with odds ratios of 1.50 (95% confidence interval [CI] 1.16 to 1.93) and 1.40 (95% CI 1.04 to 1.88) respectively; and a decreased risk of outpatient attendance, though this did not reach significance 0.91 (95% CI 0.68–1.23) – see Table 2. A physical long-term condition was significantly associated with a higher probability of utilisation across all four services. Interestingly, socioeconomic status (IMD quintile) was only significantly associated with outpatient utilisation. A full set of results across all four outcomes and including all covariates are provided in Additional file 3: Table S3.

**Table 2:** Adjusted^a^ logistic regression models: health care utilisation for the full cohort and subgroup analysis (patients aged 75 and over)

Household composition (vs other)	At least 12 general practice appointments		At least 1 emergency department attendances		At least 1 inpatient admissions		At least 5 outpatient appointments	
	Odds ratio	95% Confidence Interval	Odds ratio	95% Confidence Interval	Odds ratio	95% Confidence Interval	Odds ratio	95% Confidence Interval
Full Analysis								
Alone	1.40*	1.04–1.88	1.50*	1.16-1.93	1.30	0.99–1.70	0.91	0.68–1.23
Subgroup Analysis								
70 +: Alone	1.54*	1.06–2.24	1.62*	1.18-2.24	1.45*	1.04-2.01	0.85	0.60–1.21
75 +: Alone	1.47	0.94–2.30	1.33	0.91–1.96	1.45	0.98–2.14	0.78	0.51–1.20
80 +: Alone	1.61	0.90–2.86	1.30	0.79–2.14	1.25	0.75–2.09	0.53*	0.31-0.93

^a^Adjusted for household structure, sex, Index of Multiple Deprivation (IMD) quintile, physical long-term conditions and mental long-term conditions

*Denotes significance at *p* < 0.05

### Subgroup analysis

Results are consistent with the main analysis and show an increasing impact of living alone between the group age 65 and over and 70 and over. For general practice utilisation, patients aged 70 + living alone had a significantly higher utilisation of general practice services compared to older patients living with others, odds ratio 1.54 (95% CI 1.06–2.24). Similarly, for emergency department utilisation, patients living alone aged 70+ had a significantly higher utilisation, odds ratios 1.62 (95% CI 1.18–2.24). While the results remain consistent, the effect is no longer significant at older ages; however, this may be due to reduced sample size for this cohort – see Table 2. The exception to this trend being outpatient appointments, where in the very old (80+) living alone was associated with a statistically significant decreased utilisation 0.53 (95% CI 0.31–0.93). A potential explanation of this trend is increased usage of homebased care or social care in older age groups.

### Sensitivity analysis

The sensitivity analysis produced results consistent with the main analysis. When we examined high users of general practice appointments and used a threshold of at least 16 appointments, there was still a significant positive association between living alone and utilisation; with odds ratios of 1.73 (95% CI 1.17–2.57). Sensitivity analysis results for the outpatient analysis were consistent with those seen in the main analysis – see Table 3 and Additional file 4: Table S4.

**Table 3:** Sensitivity analysis; adjusted logistic regression models: general practice and outpatient health care utilisation, with higher thresholds

	Model			
	At least 16 general practice appointments		At least 8 outpatient appointments	
	Odds ratio	95% Confidence Interval	Odds ratio	95% Confidence Interval
Household composition (vs other)				
Alone	1.73*	1.17–2.57	0.98	0.64–1.49

*Denotes significance at *p* < 0.05

## Discussion

We analysed 1447 older patients and examined the association between living alone and four types of health care utilisation. After controlling for patient demographic and clinical characteristics, older patients living alone were 50% more likely to have an emergency department attendance and 40% more likely to have over 12 general practitioner appointments compared to older patients living with others. Given there is a growing population of older people living alone with complex health needs [1, 4] and patients living alone have higher health care services utilisation, there are substantial implications for the increasing pressure on general practice and acute care services.

The relationship between living alone and health care utilisation is more pronounced in patients aged 70 and over, and amongst the highest users of health care services. We also found that those living alone, and aged over 80 were less likely to utilise outpatient appointments. These results highlight the usefulness of data collected in electronic health records to understand non-clinical factors, such as whether a patient lives alone, when designing interventions intended to improve quality of care for patients, and reduce demand for secondary care [23].

To our knowledge our study is the first in the UK to examine this question using electronic health records, and so a direct comparison is not possible, though our findings do contradict those presented in the most comparable UK study. The existing study found that those living alone had lower general practitioner utilisation, odds ratio 0.78 (95% CI 0.6–1.0), while we show that those living alone had significantly higher general practice utilisation, odds ratio 1.40 (95% CI 1.04–1.88) [18]. However, the results presented here are consistent with international studies conducted across health care systems in Mexico, the United States and Australia. Previous research has found an association between older people who live alone and increased health care services utilisation [13–17]. In an Australian study, patients who live alone have a 2.7% higher probability of accessing inpatient services [15]; and in a study conducted in London, there is a strong correlation between older patients living alone and emergency department attendances [16]. Despite differences in the structure of health care systems across the world, the results presented in this paper are consistent with the findings of international studies and provide additional evidence that older patients living alone are higher users of general practice and emergency services.

Our study has a number of strengths; unlike many existing studies [13–16, 18], we had access to pseudonymised patient electronic health records, from both primary and secondary care and could control for demographic and clinical characteristics, including many long-term conditions. Additionally, these records contained secondary care utilisation over the last year and household-level identifiers. As a result, we did not rely on self-reported information from survey data, a drawback of many of the existing studies. [13–15, 18, 19] Furthermore, the use of the data in this manner demonstrates that general practitioners can play an active role in the use of their routine data in identifying subgroups of patients who live alone and designing interventions that are suited to their needs.

This study is limited by several weaknesses. The electronic health records were all from one urban general practice in South-East London. Their relatively young and deprived population [20], may not be representative of England as a whole. Moreover, the provision of local health care and other services for older patients may vary geographically and may not be representative of England, though data was not available to examine this. No adjustment was made for clustering by practice site as the two sites were located close to one another and are subject to the same management. The secondary care utilisation was reported to Valentine Health by local secondary care providers and may be underestimated. However, there are no local policies or practices in place where reporting is more likely to be made for patients who live alone, and this should not impact on the conclusions of the study. Furthermore, it was not possible to separate out elective and emergency inpatient admissions as the reasons for inpatient admissions were not available. Data relating to homebased health and social care were not available, as a result it is not possible to account for the level of formal support patients may be receiving through these channels. This study only covers one year of utilisation; the impact of living alone, or the lifetime socioeconomic factors associated with living alone [24], may impact the severity of disease and other social factors [25], which in turn impact on health care utilisation. As our data was limited to patients registered at Valentine Health, our households could be missing those people registered at a different practice, or not registered at all. However, we would expect all older patients to be registered at a general practice, and most likely at the same one as the people they live with.

Our results demonstrate an association between older patient’s living arrangements and their health care utilisation. While the study design does not allow us to identify a causal link, it highlights the potential to identify and target patients with higher needs who live alone. Within primary care, there is scope for general practitioners to refer older people living alone to local non-clinical services, to help support them both emotionally and practically [26, 27] or review their care to identify potential areas of unmet need such as missed outpatient appointments. Within an acute care setting, interventions implemented prior to discharge which offer additional support to patients living alone may help reduce the risk of readmissions.

While this research demonstrates an association between living alone and health care utilisation, it does not explain the mechanism driving increased utilisation of health care services for older patients living alone. Although we controlled for deprivation, there may be wider social and deprivation factors that may influence both living arrangements and ill health, therefore increasing the demand for health services. One possibility is that social isolation and a lack of social support could result in poor health, and increased health needs for older patients living alone [28]. A second possible explanation is that patients who choose to live alone may not be either lonely or socially isolated, but may require increased support during periods of ill health, and utilise acute health care services meet this need. Further research is needed to determine whether acute care is the most appropriate setting to meet these needs, or whether there are other interventions that can be designed to meet these needs and to reduce the utilisation of acute care services.

## Conclusion

Older patients living alone are 50% more likely to access emergency care services and 40% more likely to have more than 12 general practice appointments over a 12-month period, compared to the older patients not living alone. General practitioners can play an active role in identifying older patients living alone through routine data and providing additional support where required. The context of patients’ living arrangements has important implications for health care needs and the utilisation of health care services. Older patients who live alone may require additional support to support both clinical and nonclinical needs; identifying which individuals would most benefit from such support should be the target of further research.

## Additional files

Additional file 1: **Table S1.** Utilisation by utilisation, count (percentage %). This table sets out a count of patients by utilisation and demonstrates the relationship between utilisation of different health care services (DOCX 22 kb)

Additional file 2: **Table S2.** Unadjusted logistic regression models estimating health care utilisation. This table sets out the results of the unadjusted logistic regression models and the association between living alone and health care utilisation. Odds ratios and *p* values are reported. (DOCX 22 kb)

Additional file 3: **Table S3.** Adjusted logistic regression models estimating health care utilisation. This table sets out the results of the adjusted logistic regression models and the association between living alone and health care utilisation, as well as all the other factors in the model and health care utilisation. Odds ratios and *p* values are reported. (DOCX 23 kb)

Additional file 4: **Table S4.** Sensitivity testing - Logistic regression modelling health care utilisation. This table sets out the results of the sensitivity testing for general practice utilisation and outpatient attendances. Odds ratios and p values are reported. (DOCX 23 kb)
