# Supplementary material for: The association between living alone and health care utilisation in older adults: a retrospective cohort study of electronic health records from a London general practice
Source: BMC Geriatr. 2018 Dec 5;18:269. doi: 10.1186/s12877-018-0939-4 (PMC6280341; doi:10.1186/s12877-018-0939-4)
Supplement: Supplementary file 1 — Table S1. Utilisation by utilisation, count (percentage %). This table sets out a count of patients by utilisation and demonstrates the relationship between utilisation of different health care services (DOCX 22 kb) [file 12877_2018_939_MOESM1_ESM.docx]

**Appendix 1: Utilisation by utilisation, count (percentage%)**

|  | **At least 12 general practice appointments** | | **At least 1 emergency department attendance** | | **At least 1 inpatient admission** | | **At least 5 outpatient appointments** | | **Total cohort**  **(n=1,447)** |
| --- | --- | --- | --- | --- | --- | --- | --- | --- | --- |
|  | **No**  **(n=1,192)** | **Yes**  **(n=255)** | **No**  **(n=1074)** | **Yes**  **(n=373)** | **No**  **(n=1,126)** | **Yes**  **(n=321)** | **No**  **(n=1,197)** | **Yes**  **(n=250)** |  |
| At least 12 general practice appointments (%) | -- | -- | 112 (10.4%) | 143 (38.3%) | 140 (12.4%) | 115 (35.8%) | 157 (13.1%) | 98 (39.2%) | 255 (17.6%) |
| At least 1 emergency department attendances (%) | 230 (19.3%) | 143 (56.0%) | -- | -- | 136 (12.1%) | 237 (73.8%) | 268 (22.4%) | 105 (42.0%) | 373 (25.8%) |
| At least 1 inpatient admissions (%) | 206 (17.3%) | 115 (45.1%) | 84 (7.8%) | 237 (63.5%) | -- | -- | 209 (17.5%) | 112 (44.8%) | 321 (22.2%) |
| At least 5 outpatient appointments (%) | 152 (12.8%) | 98 (38.4%) | 145 (13.5%) | 105 (28.2%) | 138 (12.3%) | 112 (34.9%) | -- | -- | 250 (17.3%) |

Note: percentages may not sum to 100% due to rounding.
