# Supplementary material for: The association between living alone and health care utilisation in older adults: a retrospective cohort study of electronic health records from a London general practice
Source: BMC Geriatr. 2018 Dec 5;18:269. doi: 10.1186/s12877-018-0939-4 (PMC6280341; doi:10.1186/s12877-018-0939-4)
Supplement: Supplementary file 3 — Table S3. Adjusted logistic regression models estimating health care utilisation. This table sets out the results of the adjusted logistic regression models and the association between living alone and health care utilisation, as well as all the other factors in the model and health care utilisation. Odds ratios and p values are reported. (DOCX 23 kb) [file 12877_2018_939_MOESM3_ESM.docx]

**Appendix 3: Adjusted logistic regression models: health care utilisation for the full cohort**

|  | **At least 12 general practice appointments** | | | **At least 1 emergency department attendances** | | | | **At least 1 inpatient admissions** | | | | **At least 5 outpatient appointments** | | | |
| --- | --- | --- | --- | --- | --- | --- | --- | --- | --- | --- | --- | --- | --- | --- | --- |
|  | **Odds ratio** | **95% Confidence Interval** | | **Odds ratio** | | **95% Confidence Interval** | | **Odds ratio** | | **95% Confidence Interval** | | **Odds ratio** | | **95% Confidence Interval** | |
| Household structure (vs Other) |  |  | |  | |  | |  | |  | |  | |  | |
| Alone | 1.40* | 1.04-1.88 | | 1.50* | | 1.16-1.93 | | 1.30 | | 0.99-1.70 | | 0.91 | | 0.68-1.23 | |
| Age band (vs 65 to 69) |  |  | |  | |  | |  | |  | |  | |  | |
| 70 to 74 | 0.71 | 0.47-1.08 | | 0.72 | | 0.50-1.03 | | 0.93 | | 0.64-1.36 | | 1.14 | | 0.76-1.70 | |
| 75 to 79 | 0.80 | 0.51-1.25 | | 1.09 | | 0.75-1.59 | | 1.42 | | 0.96-2.10 | | 1.33 | | 0.86-2.04 | |
| 80 plus | 1.01 | 0.69-1.47 | | 1.39* | | 1.00-1.93 | | 1.69* | | 1.19-2.39 | | 1.21 | | 0.82-1.79 | |
| Female (vs male) | 1.62* | 1.20-2.18 | | 0.91 | | 0.71-1.17 | | 0.80 | | 0.62-1.05 | | 1.16 | | 0.86-1.56 | |
| Index of Multiple Deprivation (IMD) Quintile (vs 1) |  |  | |  | |  | |  | |  | |  | |  | |
| 2 | 1.20 | 0.75-1.92 | | 0.94 | | 0.63-1.39 | | 0.80 | | 0.53-1.22 | | 1.42 | | 0.86-2.34 | |
| 3 | 1.26 | 0.78-2.04 | | 0.84 | | 0.56-1.25 | | 1.01 | | 0.67-1.54 | | 1.77* | | 1.07-2.93 | |
| 4 | 1.67* | 1.06-2.65 | | 0.95 | | 0.64-1.41 | | 0.91 | | 0.60-1.38 | | 1.69* | | 1.03-2.77 | |
| 5 | 0.99 | 0.62-1.58 | | 0.71 | | 0.48-1.04 | | 0.78 | | 0.52-1.15 | | 1.95* | | 1.22-3.12 | |
| Physical long-term conditions (+1 from mean) | 1.54* | 1.41-1.68 | | 1.40* | | 1.29-1.51 | | 1.43* | | 1.32-1.55 | | 1.56* | | 1.43-1.70 | |
| Mental long-term condition (vs not) | 1.63* | 1.19-2.24 | | 1.69* | | 1.27-2.24 | | 1.41* | | 1.04-1.90 | | 1.05 | | 0.74-1.47 | |
| *Denotes significance at p<0.05 |  | |  | |  | |  | |  | |  | |  | |  |
