# Supplementary material for: The association between living alone and health care utilisation in older adults: a retrospective cohort study of electronic health records from a London general practice
Source: BMC Geriatr. 2018 Dec 5;18:269. doi: 10.1186/s12877-018-0939-4 (PMC6280341; doi:10.1186/s12877-018-0939-4)
Supplement: Supplementary file 4 — Table S4. Sensitivity testing - Logistic regression modelling health care utilisation. This table sets out the results of the sensitivity testing for general practice utilisation and outpatient attendances. Odds ratios and p values are reported. (DOCX 23 kb) [file 12877_2018_939_MOESM4_ESM.docx]

**Appendix 4: Sensitivity testing**

|  | **At least 16 general practice appointments** | | | **At least 8 outpatient appointments** | | | |
| --- | --- | --- | --- | --- | --- | --- | --- |
|  | **Odds ratio** | **95% Confidence Interval** | | **Odds ratio** | | **95% Confidence Interval** | |
| Household structure (vs Other) |  |  | |  | |  | |
| Alone | 1.73* | 1.17-2.57 | | 0.98 | | 0.64-1.49 | |
| Age band (vs 65 to 69) |  |  | |  | |  | |
| 70 to 74 | 1.10 | 0.65-1.87 | | 1.20 | | 0.69-2.08 | |
| 75 to 79 | 0.90 | 0.49-1.65 | | 1.14 | | 0.62-2.11 | |
| 80 plus | 1.37 | 0.84-2.22 | | 0.78 | | 0.44-1.41 | |
| Female (vs male) | 1.47 | 0.99-2.16 | | 1.03 | | 0.67-1.56 | |
| Index of Multiple Deprivation (IMD) Quintile (vs 1) |  |  | |  | |  | |
| 2 | 1.46 | 0.79-2.71 | | 0.72 | | 0.36-1.44 | |
| 3 | 1.21 | 0.63-2.34 | | 1.32 | | 0.69-2.50 | |
| 4 | 1.80 | 0.98-3.32 | | 0.85 | | 0.43-1.70 | |
| 5 | 1.42 | 0.77-2.60 | | 1.07 | | 0.57-1.99 | |
| Physical long-term conditions (+1 from mean) | 1.54* | 1.39-1.71 | | 1.50* | | 1.34-1.68 | |
| Mental long-term condition (vs not) | 1.57* | 1.05-2.33 | | 0.93 | | 0.57-1.52 | |
| *Denotes significance at p<0.05. |  | |  | |  | |  |
